# Gene3D: Multi-domain annotations for protein sequence and comparative genome analysis

**DOI:** 10.1093/nar/gkt1205

**Published:** 2013-11-21

**Authors:** Jonathan G. Lees, David Lee, Romain A. Studer, Natalie L. Dawson, Ian Sillitoe, Sayoni Das, Corin Yeats, Benoit H. Dessailly, Robert Rentzsch, Christine A. Orengo

**Affiliations:** ^1^Division of Biosciences, Institute of Structural and Molecular Biology, University College London, Gower Street, London WC1E 6BT, UK, ^2^Department of Infectious Disease Epidemiology, Imperial College London, St Mary's Campus, Norfolk Place, London W2 1PG, UK and ^3^Robert Koch Institut, Research Group Bioinformatics Ng4, Nordufer 20, 13353 Berlin, Germany

## Abstract

Gene3D (http://gene3d.biochem.ucl.ac.uk) is a database of protein domain structure annotations for protein sequences. Domains are predicted using a library of profile HMMs from 2738 CATH superfamilies. Gene3D assigns domain annotations to Ensembl and UniProt sequence sets including >6000 cellular genomes and >20 million unique protein sequences. This represents an increase of 45% in the number of protein sequences since our last publication. Thanks to improvements in the underlying data and pipeline, we see large increases in the domain coverage of sequences. We have expanded this coverage by integrating Pfam and SUPERFAMILY domain annotations, and we now resolve domain overlaps to provide highly comprehensive composite multi-domain architectures. To make these data more accessible for comparative genome analyses, we have developed novel search algorithms for searching genomes to identify related multi-domain architectures. In addition to providing domain family annotations, we have now developed a pipeline for 3D homology modelling of domains in Gene3D. This has been applied to the human genome and will be rolled out to other major organisms over the next year.

## INTRODUCTION

Proteins commonly contain ≥1 discrete independently folding domains. Similar to these folded domains, it is becoming increasingly clear that many proteins can also contain long disordered sections that are also of functional importance. The CATH database which focuses on the folded domain, classifies structures in the PDB into their constituent domains, and each domain is subsequently assigned to a single superfamily by homology (1,2). The classified domain structure sequences are used in a pipeline to build domain superfamily specific HMMs that are then used to identify domains in structurally uncharacterized protein sequences. As some CATH superfamilies can be large and functionally diverse, we recently introduced protocols for subdividing each superfamily into functional sub-families known as FunFams (2,3). Owing to the speed and scale at which domain annotations can be assigned to sequences, the computational assignment of functional family domains to proteins is a powerful tool for bridging the gap in functional coverage of the ever-expanding genome and sequence databases. While more development is needed to improve accuracy, the CATH-FunFams were the top performing domain-based method for assigning protein functions in a recent international competition (4). After the domains have been assigned to a protein, DomainFinder (5) is used to resolve issues of overlapping domain assignments to derive a single combination of domains known as the multi-domain architecture (MDA). To increase the utility of the domain annotations in Gene3D, we integrate many other complementary data sources including UniProt (6), UniProt-GO (7), NCBI taxonomy (8), DrugBank (9) and OMIM (10). Other domain annotation resources include Pfam(11) and SUPERFAMILY(12). SUPERFAMILY is more similar to Gene3D in that it makes use of the structural domains from SCOP as its starting source of established domains. SUPERFAMILY adds a small percentage of annotations for structural superfamilies classified in SCOP but not in CATH. A new resource, Genome3D (13), provides a consensus assignment showing where Gene3D and SUPERFAMILY agree on matches to a particular domain within a protein sequence.

**Figure 1.**
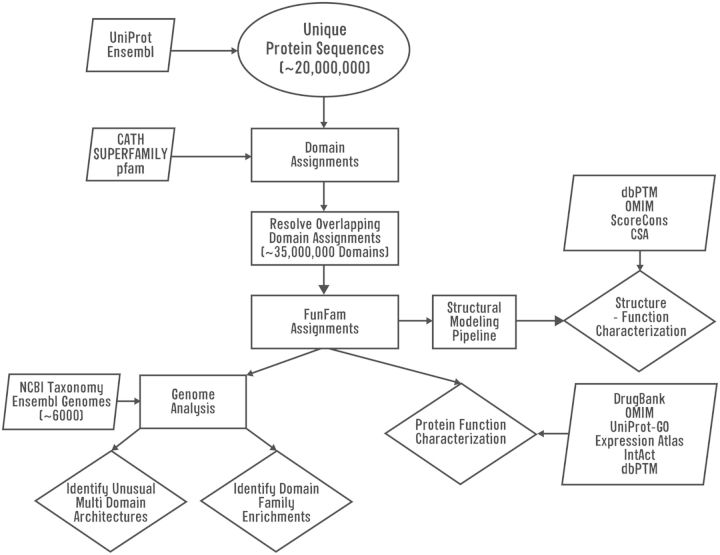
Workflow of the Gene3D pipeline from data input from external resources (Parallelogram shaped boxes) to useful functions for the user (Diamond shaped boxes). Rectangular boxes represent data processing steps.

As a summary of the principal updates for this article, we have expanded Gene3D domain assignments considerably by using CATH version 4.0 (updated from CATH version 3.5). As CATH version 4.0 contains nearly 50% more domain structures this has resulted in greater sequence coverage. We have also updated our core sequence and genome databases, increasing the number of unique sequences to >20 million. The FunFams have been updated using the new CATH version 4.0 superfamily assignments for Gene3D version 12. To provide 3D models for selected model organisms, we have developed a homology modelling pipeline that exploits the FunFam multiple sequence alignments to improve accuracy and coverage. For the first time in Gene3D, we have fully integrated Pfam (11) and SUPERFAMILY (12) domain family assignments by using DomainFinder to give more complete MDA assignments. We have also included a comprehensive data set of post translational modifications (PTMs) (14). For an overview of the Gene3D data pipeline see Figure 1. The website has also been updated to provide improved analysis tools, including a sequence search tool to find proteins with similar MDAs.

### New data and features in Gene3D

#### Significant increase in numbers of sequences annotated with at least one domain

The number of sequences in the database has increased by 45% since our last publication with >6000 cellular genomes now present. We report a significant increase in domain coverage of sequences in Gene3D, over the previous NAR database publication (15). The new release, Gene3D version 12, has 25 615 754 CATH domains assigned for 21 662 155 distinct protein sequences, belonging to 2738 CATH superfamilies. On the set of Ensembl genome sequences common to the previous and current releases, there is a substantial increase in domain coverage from 60 to 64% of sequences assigned at least one CATH domain. Some branches of life show greater increases, with metazoan species showing an 8.7% increase in the proportion of sequences assigned at least one domain. The increase in coverage can partly be ascribed to the increase in PDB domains assigned by the CATH domain assignment protocol and partly to changing the CATH domain HMM model building method from target2K to jackhmmer. We now include domains from Pfam-A, Pfam-B and SUPERFAMILY, which are integrated using the DomainFinder algorithm (5). This method uses graph theory to identify the optimal combination of domain family matches, having acceptable scores, to maximize coverage of the sequence and remove domain overlaps. After integration, this produces 35 253 067 domain assignments with no sequence overlaps between them. On the Ensembl genomes sequence set the addition of Pfam-A (11), Pfam-B and SUPERFAMILY(12) increase the coverage from 64 to 84% showing that the different domain-based resources are highly complementary.

In our previous Gene3D publication (15), we introduced more specific domain assignments based on functional sub-classification of CATH superfamily assignments (FunFams). FunFams are built by agglomerative clustering of Gene3D domain sequences using the E-value returned from profile–profile comparisons (16). Since >50% of sequences in Gene3D belong to functionally diverse superfamilies, and since the FunFams are functionally cohesive families, using FunFam-based assignments allows us to provide more reliable functional annotation than using assignments from broad, highly diverse CATH superfamilies. The FunFams have been benchmarked against manually curated, experimentally annotated functional classifications, such as the SFLD (17) and more recently have been shown to be highly competitive in the international CAFA functional annotation assessment (4).

#### New MDA comparison method

**Figure 2.**
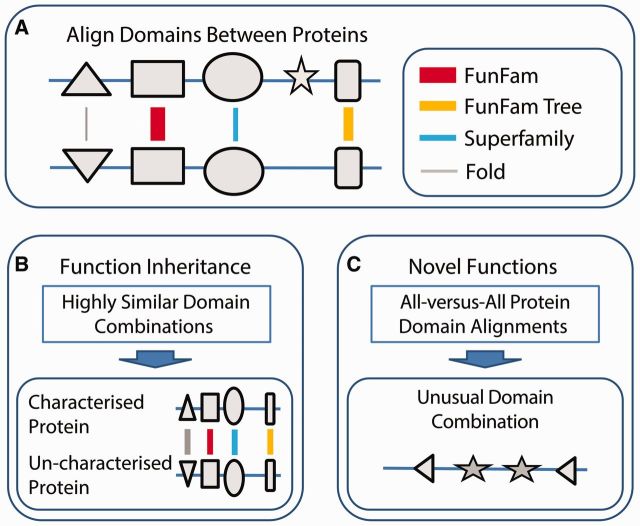
The MDA alignment method in Gene3D uses the Needleman–Wunsch (NW) algorithm. (**A**) Domain matches in the substitution matrix for the NW algorithm can take place at multiple levels. The highest scoring match is the FunFam level (FunFam Match). The next highest scoring match is between different FunFams from the same superfamily scored by their similarity in a hierarchical tree of FunFams built from profile–profile comparisons (FunFam-Tree Match). The next highest scoring match is at the homologous superfamily level (Superfamily Match). Finally, domains with the same fold can also contribute a positive similarity score in the domain alignment (Fold Match). (**B**) Domain alignments can be used to find functionally similar proteins by identifying proteins with a similar MDA. (**C**) All versus All MDA alignments have been carried out to identify those proteins with distinctive domain combinations in a genome (C).

As mentioned earlier in the text, Gene3D now incorporates FunFams, CATH, Pfam and SUPERFAMILY domain assignments to produce highly comprehensive MDAs. We have added in a new inter genome domain architecture similarity search algorithm. This method uses the Needleman–Wunsch (NW) dynamic programming algorithm, modified to align domain strings (MDA) between two proteins (instead of more usual protein sequence strings) (Figure 2). Aligning domain architectures between two proteins allows us to find proteins with a similar ‘domain grammar’ (and hence molecular function). This approach has similarities to methods described previously (18). However, our method also allows domain matches in the resulting alignment (as opposed to gaps) to occur at multiple levels in the domain family hierarchy. The most specific matches in the alignment are between identical FunFams (followed by similar FunFams based on FunFam hierarchical trees). The next most specific matches are between identical domain families that imply shared homology but not necessarily function (such as the homologous superfamily level in CATH). Finally, we also allow matches between domains without identifiable homology but with the same arrangement of secondary structures, known as the protein fold (i.e. the T or topology level in CATH). Each type of match is given a positive score in the substitution matrix used by the NW algorithm, with the score increasing for more specific matches (i.e. same fold > same homologous superfamily > FunFam tree > same FunFam) (Figure 2A). In the alignment matrix, mismatches are scored as −1.0 and gaps are penalized with a score of −0.01. Hence, if two proteins produce highly similar FunFam domain alignments, it provides some evidence of functional similarity (Figure 2B). We have pre-calculated all-versus-all MDA similarities for the proteins in eukaryotic Ensembl genomes. The MDAs that have low similarity to any others in comparison genomes can be considered distinctive for that genome (and potentially carry out interesting lineage specific functions) (Figure 2C).

#### 3D structural models provided for selected organisms

Another advantage of the FunFams is that as well as being functionally more pure than CATH superfamily clusters, they also represent structurally cohesive clusters that can be used for homology modelling. Analysis has shown that we can build significantly more 3D models, at a certain high level of accuracy, than by using simple pairwise thresholds on sequence similarity i.e. building models for target sequences sharing ≥30% sequence identity with a protein of known structure (16). Therefore, we have used the FunFams in a new pipeline (FF-mod) to provide structural models for a subset of genomes. For a specific target sequence, FF-mod uses BLASTp (19) to identify the best structural template(s) within the same FunFam of the target sequence. We select up to five templates. The target sequence and the template(s) are then extracted and realigned using MAFFT (20) with the L-INS-i mode for maximum accuracy. They are finally put into a homology modelling framework using Modeller as the core engine (21). The insertions are discarded from the modelling when they are >10 residues long. One hundred models are generated and the best one, according to its DOPE score, is retained. The residue numbering in the model domain corresponds to the numbering in the whole protein, facilitating further annotations. We have applied this pipeline to the human Ensembl genome, and we are in the process of extending this to genomes of other model organisms (e.g. fly, yeast and mouse). For the human genome, we have also identified highly conserved residue positions for a given FunFam by running the Scorecons algorithm (22) on the MAFFT alignments of the FunFam core sequence set. The core set includes all sequences with a Gene Ontology (GO) experimental annotation and all sequences of known structure.

#### New sequence annotations on PTMs

As another new feature in Gene3D database, we now integrate PTMs from the Database of Post translational modifications [dbPTM (14)]. This information can be valuable when comparing the MDAs between proteins as it can be informative to consider the position of the PTMs in the MDAs, particularly when a domain has a structure/structural model associated with it and there is information on conserved sequence positions.

### Website Updates

The website has been redesigned to provide better and more unique analysis tools. For brief descriptions of each page see the following sections.

#### Individual protein and domain pages

##### MDA assignments

The principal results on an individual protein page are the domain assignments which show the MDA with no overlapping domain assignments. Among other things the Protein View page shows details of predicted functions, OMIM mutations (10), known drugs and physical protein interactions. We display MDA representations aligned with the protein sequence using the BioJS sequence library (23). We also provide domain architecture images, which can be viewed in compact or normal views where the domain image areas or lengths are proportional to the number of residues in the domain, respectively. The compact view helps to visualize small domains in MDAs with several large domains. We have added a search form to find proteins from a specified genome with identical or similar MDAs.

##### Structural models

**Figure 3.**
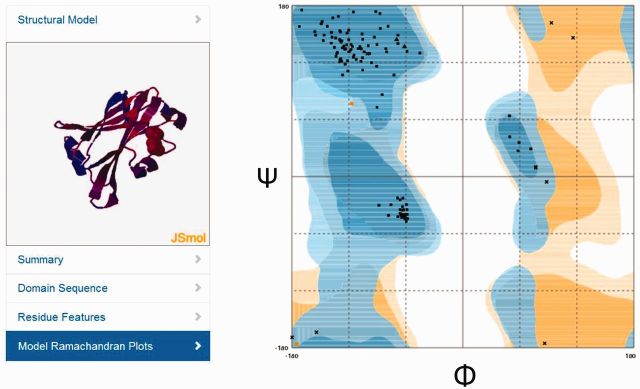
Individual domain summary page (example is the C-terminal domain of human siah2,SIAH2_HUMAN) showing a modelled structure along with the Ramachandran plot from the Rampage software package (25) used as part of the quality control step. Residues in the sequence and structure are coloured by conservation across the FunFam (blue->red indicates increasing conservation).

From the Protein page, it is possible to link out to the individual domain sequence pages (Figure 3). Structural models can be displayed using JSmol, which removes the need to install Java as JSmol uses HTML5. The structural models provide a means of visualizing the locations of sequence features such as PTMs (14), OMIM (10) and Eukaryotic Linear Motifs (ELMs) (24). To help in quality control of the modelled structure, we provide, DOPE profiles obtained from Modeller itself, the multiple sequence alignments used by Modeller and Ramachandran plots generated by Rampage (25). The Rampage output provides details on the expected and actual percentages of residues in allowed, favoured and disallowed regions of the Ramachandran map.

The structural data and sequence conservation information can be useful both for functional interpretation and for increasing the confidence in various sequence features. For example most ELMs must be exposed on the surface to be functional. From the FunFam alignment of the modelled domain it is possible to colour residues in the structure based on their conservation. In a similar manner, it is possible to colour residues according to the number of phosphorylation sites that have been identified at a given point in the alignment across all members of the alignment. In the near future, we will be adding complementary data on known interactions and the Catalytic Site Atlas for displaying on the structure.

##### Function prediction

From the GO terms associated with each FunFam, we are able to annotate the putative functions of a protein as a whole. This provides a high-throughput means of protein functional annotation. Sometimes the GO predictions from FunFams are extensive, so we provide links to visualize the results using the Revigo resource (26).

##### Protein–Protein interactions

We provide details on the physical protein interactions for all organisms from IntAct (27). The interactions are restricted to direct-physical by filtering on the PSI-MI (28) code MI:0407, and all its child terms. The networks are visualized in Cytoscapeweb.js thus removing the dependency on flash for displaying networks in the previous version of Gene3D. Domain families assigned to the proteins of the network are also displayed as nodes. A link in the network between a protein and domain family indicates the protein contains that domain family.

### Other Updates

We have improved our genome summary pages to better identify the most highly enriched domain families for a given genome by using the Fisher's exact test. The enrichments are calculated relative to the closest set of genomes as judged by the NCBI taxonomy. We also provide a list of the most distinct MDAs for a given genome using the alignment methods described earlier in the text.

The average MDA length (the number of domains assigned to a protein) varies greatly between different taxonomic levels, and we now provide the MDA length distribution plots in the summary page. Where multiple transcripts are assigned to a gene we select the longest transcript.

Apart from a genome summary, it is also possible for the user to input a higher taxonomic level ID to get similar summary pages. In this case, the NCBI taxonomy is used to group all genomes at the specified taxonomic level before producing summary outputs. In this way, it is possible to get summaries for groups such as Metazoa.

For the genome comparison pages, we identify domain families with significant differences in gene counts. We also provide information on those MDAs that are most distinctive between the genomes, both in terms of MDA alignment distances and unique domain compositions. We have also updated our genome comparison tool, so any two taxonomic levels can be easily compared.

For a domain family summary page, we provide information on how the family is distributed across the Tree of Life and at which points in the taxonomic tree the family shows expansions in the number of genes in which it occurs. We also provide a table detailing the list of MDAs in which the domain family is found. If the MDA has a known Drug or reliable UniProt-GO annotation, then this data is displayed. Further details on the domain family e.g. structural variation of relatives are available from the equivalent pages at the CATH resource.

### Data Downloads

We provide the same downloads as for previous releases at the usual ftp site (ftp://ftp.biochem.ucl.ac.uk/pub/gene3d_data/CURRENT_RELEASE/). We have added the fully resolved MDAs Gene3D_12_MDAs.gz. The domain assignments for the Ensembl genomes can be found at Gene3D_12_MDA-genomes.gz.

## DISCUSSION

Gene3D continues to cope with the challenges faced by the ever-expanding sequence data sets and uniquely provides CATH structural family and FunFam functional family domain assignments for all UniProt and Ensembl sequences. The number of sequences in the database has increased by 45% since our last publication. Despite this, we have seen a progressive increase in sequence coverage of 8% over the past two releases. The addition of Pfam-A, Pfam-B and SUPERFAMILY increase the coverage by a further 20% showing the great value of combining this data. This increase in coverage shows that most sequences can be divided into a small subset of domain families. This phenomenon is robust, as the level of domain coverage has increased despite the concurrent increase in the number and diversity of sequenced genomes. Gene3D continues to harness this data through powerful sequence annotation tools and pipelines that integrate domain assignments from multiple resources to provide comprehensive Multi-Domain Architectures.

## FUNDING

IMI, EU (to J.G.L.); Wellcome Trust (to I.S.); BBSRC (to S.D.); MRC (to N.L.D.); Fondation du 450ème anniversaire de l’Université de Lausanne; and Swiss National Science Foundation [132476 and 136477 to R.A.S.]; This project has been funded in whole or in part with Federal funds from the National Institute of Allergy and Infectious Diseases, National Institutes of Health, Department of Health and Human Services [HHSN272200700058C and HHSN272201200026C to B.H.D. and C.Y.]. Funding for open access charge: Equally contributed by the Wellcome Trust and the MRC.

*Conflict of interest statement*. None declared.
